# *Pseudomonas eucalypticola* sp. nov., a producer of antifungal agents isolated from *Eucalyptus dunnii* leaves

**DOI:** 10.1038/s41598-021-82682-7

**Published:** 2021-02-04

**Authors:** Yujing Liu, Zhang Song, Hualong Zeng, Meng Lu, Weiyao Zhu, Xiaoting Wang, Xinkun Lian, Qinghua Zhang

**Affiliations:** Institute of Forest Protection in Forestry College of Fujian Agriculture and Forestry University, Fuzhou, 350002 China

**Keywords:** Bacteria, Forestry

## Abstract

*Pseudomonas* are ubiquitously occurring microorganisms and are known for their ability to produce antimicrobials. An endophytic bacterial strain NP-1^ T^, isolated from *Eucalyptus dunnii* leaves, exhibits antifungal properties against five tested phytopathogenic fungi. The strain is a Gram-negative rod-shaped bacterium containing a single polar flagellum. It is strictly aerobic, grows at 4–37 °C, 2–5% NaCl, and pH 3–7. The 16S rRNA sequence analysis showed that NP-1^ T^ belongs to the *Pseudomonas* genus. Phylogenetic analysis based on four concatenated partial genes (16S rDNA, *gyrB*, *rpoB* and *rpoD*) and the phylogenomic tree indicated that NP-1^ T^ belongs to *Pseudomonas fluorescens* lineage but is distinct from any known *Pseudomonas* species. The G + C mol % of NP-1^ T^ genome is 63.96, and the differences between NP-1^ T^ and related species are larger than 1. The digital DNA-DNA hybridization and tetranucleotide signatures are 23.8 and 0.97, which clearly separates strain NP-1^ T^ from its closest neighbours, *Pseudomonas coleopterorum* and *Pseudomonas rhizosphaerae*. Its phenotypic and chemotaxonomic features confirmed its differentiation from related taxa. The results from this polyphasic approach support the classification of NP-1^ T^ as a novel species of *Pseudomonas*, and the name of *Pseudomonas eucalypticola* is thus proposed for this strain, whose type is NP-1^ T^ (= CCTCC M2018494^T^ = JCM 33572^ T^).

## Introduction

The genus *Pseudomonas* belongs to the family Pseudomonadaceae within γ-Proteobacteria and is composed of Gram-negative, aerobic, non-spore forming rod-shaped bacteria that are motile by polar flagella^1^. *Pseudomonas* was originally described by Migula (1894) and currently comprises very heterogeneous species. New species belonging to the genus are continuously isolated from a variety of natural ecological niches, including the plant endosphere^1–3^. At the time of the writing of this manuscript, more than 254 valid species names are listed by LPSN (http://www.bacterio.net/pseudomonas.html)^4^. Due to species diversity, for the identification of each new species of *Pseudomonas*, phenotypical and chemotaxonomical analyses, multilocus sequence analysis (MLSA), and genomic comparisons should be performed^5–9^.

Some *Pseudomonas* species are well known for their pathogenicity to plants, animals or humans^10^, and several members are used for bioremediation and as biocontrol agents^11^. Many strains of *P. fluorescens*, *P. aeruginosa*, *P. chlororaphis* and *P. putida* have been used to control soil-borne plant disease because these strains can synthesize antimicrobial compounds such as phenazine, pyrrolnitrin or pyoluteorin^12–14^. During our search for biocontrol agents against *Calonectria pseudoreteaudii*, which is the pathogen of Eucalyptus leaf blight, endophytes of *Eucalyptus dunnii* leaves were isolated and selected for *C. pseudoreteaudii* inhibition tests. One bacterial isolate NP-1^ T^ could inhibit *C. pseudoreteaudii* mycelial growth, and the culture filtrate of NP-1^ T^ was capable of controlling this plant disease in vitro. In order to evaluate the biocontrol potential of the endophytic bacterial strain NP-1^ T^, its classification position and antifungal activity were determined in this study.

Strain NP-1^ T^ was identified as a member of the genus *Pseudomonas* based on its phenotypic features and 16S rDNA sequence but did not match any known species of the genus *Pseudomonas*. In the present work, a polyphasic taxonomic analysis of NP-1^ T^ was performed. A new *Pseudomonas* species with the strain NP-1^ T^ (= CCTCC M2018494^T^ = JCM 33572^ T^) as the type strain was thus proposed. Antifungal tests revealed that, NP-1^ T^ inhibited five tested phytopathogenic fungal species, which shows that NP-1^ T^ has a broad antimicrobial spectrum.

## Results and discussion

### Phylogenetic analysis

**Figure 1:**
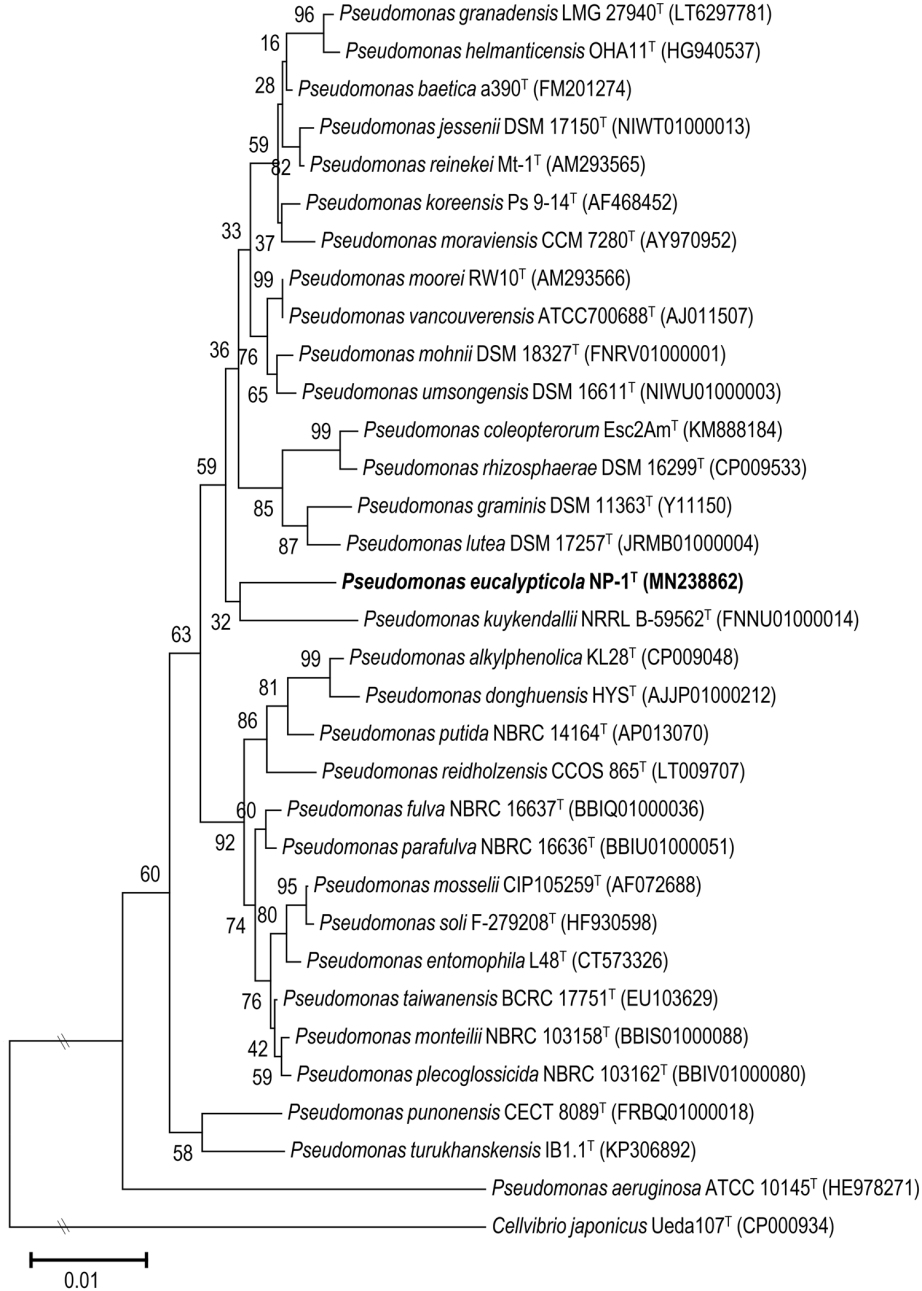
Neighbor-joining phylogenetic tree based on the 16S rRNA gene of *Pseudomonas eucalypticola* NP-1^T^ and phylogenetically close members of *Pseudomonas*. The evolutionary distances were computed using the Jukes-Cantor method. The optimal tree with a sum of branch length = 0.23535266 is shown. The percentage of replicate trees in which the associated taxa clustered together in the bootstrap test (1000 replicates) is shown next to the branches. *Cellvibrio japonicus* Ueda107^T^ was used as outgroup.

A 1444 bp fragment of the 16S rRNA gene was amplified from the *P. eucalypticola* strain NP-1^ T^, sequenced and the sequence deposited in GenBank under accession number MN 238,862. A similarity search with this sequence was performed using EzBioCloud. Thirty valid species belonging to *P*. *fluorescens* intrageneric group (IG) proposed by Mulet et al.^15^ exhibited at least 97% similarity with NP-1^ T^, and these include *P*. *vancouverensis* ATCC 700688^ T^ (98.8% similarity), *P*. *moorei* DSM12647^T^ (98.8% similarity), *P*. *koreensis* Ps9-14^ T^ (98.8% similarity), *P*. *parafulva* NBRC16636^T^ (98.5% similarity) and *P*. *reinekei* Mt-1^ T^ (98.5% similarity). The similarities with the other 25 species are provided in Supplementary Table S1. A phylogenetic tree based on the 16S rRNA sequence was constructed and is shown in Fig. 1. Strain NP-1^ T^ forms a weakly supported cluster with *P. kuykendallii* NRRL B-59562^ T^, but both strains are situated on separate branches. Strain NP-1^ T^ grouped in none known group or subgroup within *P. fluorescens* lineage, and it clusters of the outer edge of a much larger group containing several *Pseudomonas* groups/subgroups. However, *Pseudomonas* species cannot be identified based only on 16S rRNA analysis.

**Figure 2:**
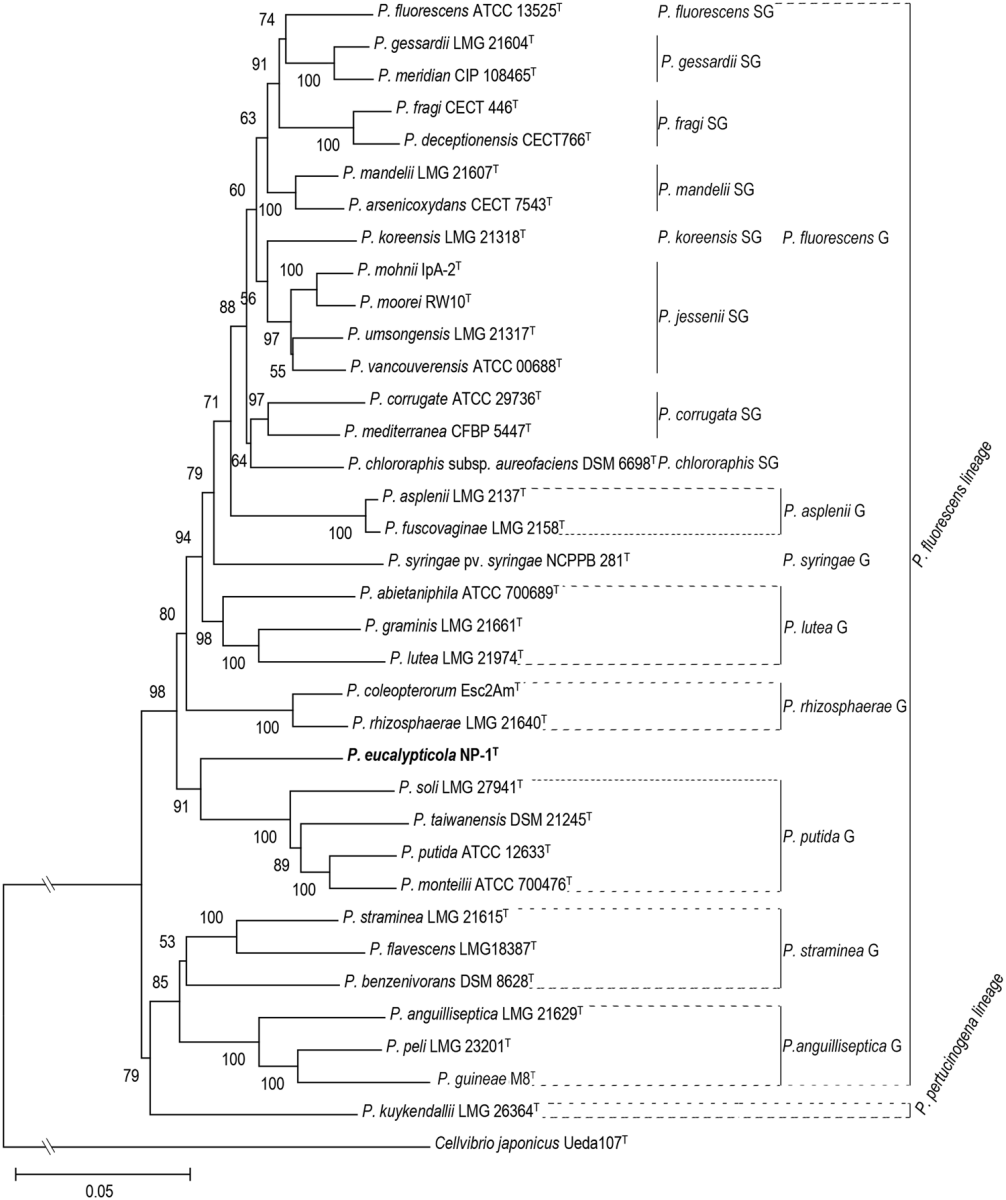
Neighbor-joining phylogenetic tree based on concatenated 16S rRNA, gyrB, rpoB and rpoD gene partial of *Pseudomonas eucalypticola* NP-1^T^ and the type strains of other *Pseudomonas* species. The evolutionary distances were computed using the Jukes-Cantor method. The evolutionary distances were computed using the Jukes-Cantor method e optimal tree with the sum of branch length = 1.37677586 is shown. The percentage of replicate trees in which the associated taxa clustered together in the bootstrap test (1000 replicates) is shown next to the branches.

The MLSA approach based on the concatenated sequences of the partial 16S rRNA, *gyrB*, *rpoB* and *rpoD* genes, has been demonstrated to greatly facilitate the identification of new *Pseudomonas* strains^16^. According to the 16S rRNA alignment, 33 species from *P*. *fluorescens* IG and one species from *P. pertucinogena* IG were selected for MLSA. The concatenated sequences of the type strains of each selected species comprised a total of 3813 bp (Supplementary Table S2) and were used for phylogenetic tree construction. The analysis of concatenated gene sequences indicated that strain NP-1^ T^ belongs to the *P. fluorescens* lineage, and this finding was supported by a bootstrap value of 91% (Fig. 2).However, NP-1^ T^ still cannot be determined which group belongs to^17^.

**Figure 3:**
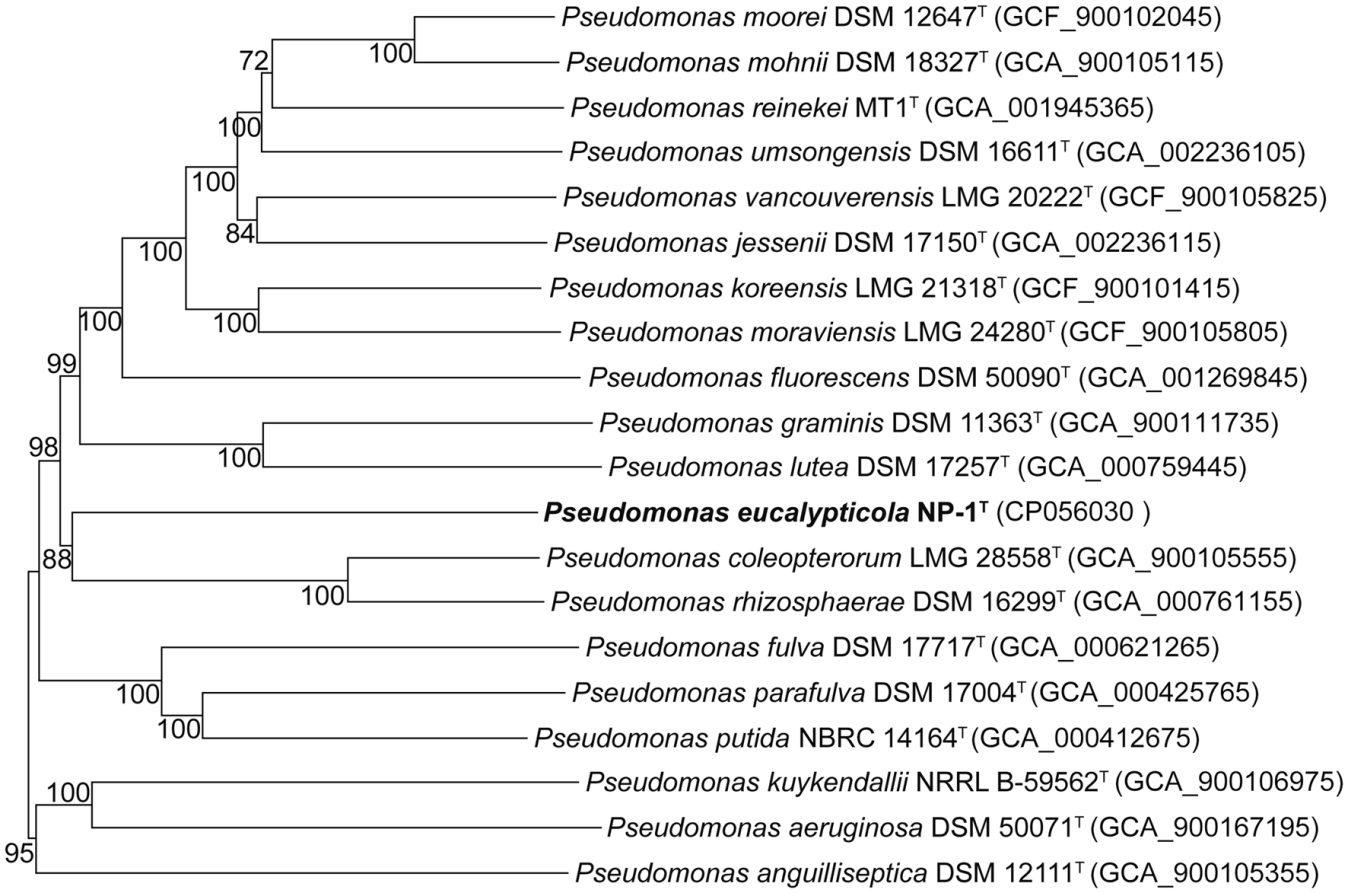
Phylogenomic tree of strain NP-1^T^ and related type strains of the genus *Pseudomonas* available on the TYGS database. The tree inferred with FastME 2.1.6.1 based on GBDP distances calculated from the genome sequences. The branch lengths are scaled in terms of the GBDP distance formula *d*_*5*_. The numbers above the branches show the GBDP pseudo-bootstrap support values > 60% from 100 replications, and the average branch support is 94.6%.

For further identification of NP-1^ T^, a phylogenomic tree inferred with GBDP was constructed by using Type (Strain) Genome Server (TYGS)^18^, and all reference type strains and their genome sources are listed in Supplementary Table S3. The result showed the presence of an independent branch supported by a bootstrap value of 88% that can be differentiated from the other *Pseudomonas* species type strains (Fig. 3) and revealed that NP-1^ T^ clustered with *P. coleopterorum* LMG 28558^ T^ and *P. rhizosphaerae* LMG 21640^ T^ which affiliated with *P. fluorescens* IG, but does not belong to any group. Strain NP-1^ T^ was not be affiliated with any previously described *Pseudomonas* species and can thus be considered to represent a novel species. Based on above-described the results, *P. coleopterorum*, *P. rhizosphaerae, P. graminis* and *P. lutea* were selected for further analysis with NP-1^ T^.

### General taxonomic genome feature

The draft genome assembly of strain NP-1^ T^ contains 6,401,699 bp. The genome of NP-1^ T^, which consists of one chromosome and one plasmid, has been deposited in GenBank under the accession numbers CP056030 and CP056031, respectively. The genome has a G + C content of 63.96 mol%, as determined from the complete genome sequence, and 83.45% of the genome is coding and consists of 5,788 genes. The similarity of the genome of *P. eucalypticola* NP-1^ T^ to other publicly available genomes of closely related *Pseudomonas* species was determined using ANI, digital DDH and G + C mol %^5–9^. Each of these comparisons yielded different ANIm and ANIb values, but the highest ANIb and ANIm values of 78.7 and 86.5 were obtained for NP-1^ T^ and *P. rhizosphaerae* LMG 21640^ T^. The similarity between *P. coleopterorum* LMG 28558^ T^ and NP-1^ T^ was higher than that between *P. graminis* DSM 11363^ T^ and *P. lutea* LMG 21974^ T^ (Table 1). All ANIb and ANIm values obtained from the comparisons of NP-1^ T^ with the other tested species were below 95%, which confirmed that strain NP-1^ T^ belongs to an independent species. The TETRA frequencies between NP-1^ T^ and the other tested type strains were lower than 0.99, which is the recommended cutoff value for species (Table 2). The digital DNA-DNA hybridization (dDDH) comparison with the draft genome of the type strain NP-1^ T^ yielded low percentages (< 30%) with all tested species (Table 2, the same species share at least70% in silico DDH). The G + C mol % differences between NP-1^T^and related species were higher than 1 (Table 2). These results, together with the ANI, and DDH values, confirm that the NP-1^ T^ strain represents a novel species in the genus *Pseudomonas*.

**Table 1:** Average nucleotide identity percentages based on BLAST (ANIb) and on MUMmer (ANIm) of *Pseudomonas eucalypticola* NP-1^T^ and type strains of closely related species of the genus *Pseudomonas*. Taxa indicated as: 1, *Pseudomonas eucalypticola* NP-1^T^; 2, *P. coleopterorum* LMG28558^T^; 3, *P. graminis* DSM11363^T^; 4, *P. lutea* LMG21974^T^; and 5, *P. rhizosphaerae* LMG21640^T^.

ANIb values	ANIm values				
	1	2	3	4	5
NP-1^T^		85.4	85.1	82.9	86.1
*P. coleopterorum* LMG 28558^T^	77.9		85.1	82.6	92.1
*P. graminis* DSM 11363^T^	76.6	76.6		82.2	85.0
*P. lutea* LMG 21974^T^	70.9	70.8	70.6		82.6
*P. rhizosphaerae* LMG 21640^T^	78.7	90.9	76.9	70.9

**Table 2:** Genome-based DDH and GC mol % difference between *Pseudomonas eucalypticola* NP-1^T^ and the type strains of closely related Pseudomonas species.

NP-1^T^ *vs*	Accession number	TETAR	DDH (%)	GC mol % difference
*P. coleopterorum* LMG 28558^T^	FNTZ00000000	0.97	23.7	1.09
*P. graminis* DSM 11363^T^	FOHW00000000	0.91	22.3	2.86
*P. lutea* LMG 21974^T^	FOEV00000000	0.69	20.0	7.82
*P. rhizosphaerae* LMG 21640^T^	CP009533	0.96	23.8	1.13

### Morphology and phenotypic characteristics

**Figure 4:**
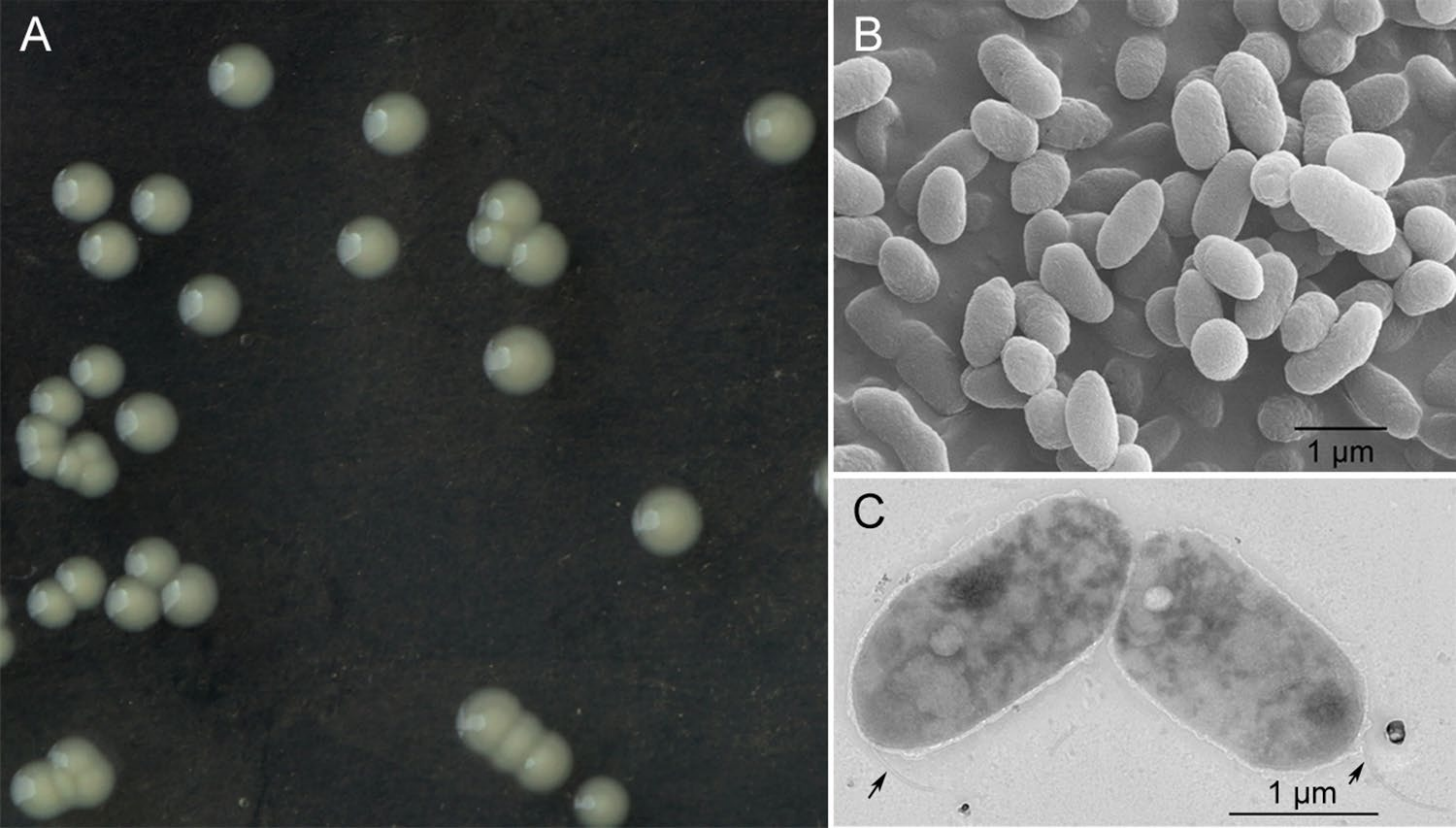
Colonies of *Pseudomonas eucalypticola* NP-1^T^ grown on LA medium at 25 °C for 48 h (A). The bacterial morphology was obtained by scanning electron microscopy (B), and the transmission electron microscopy showed the presence of a single polar flagellum (C, black arrows). Bar = 1 μm.

The colonies were round and beige with smooth surfaces and edges after incubation on LA medium for 48 h at 25 °C (Fig. 4A). The cells of NP-1^ T^ were Gram-negative (Supplementary Fig. S1), rod-shaped (1.0 μm wide, 2.0 μm longth averages), and motile due to the presence of a single polar flagellum, as observed by transmission electron microscopy (TEM, Fig. 4B and C). NP-1^ T^ grow at temperatures between 4 and 37 °C, although 25 °C was found to be the optimal temperature for growth, and no growth was detected at 42 °C. In addition, growth was observed on LB medium in the presence of 0–2% NaCl (optimum 0.5%), and at pH values of 3.0–7.0 (optimal at pH 6) (Table 3). Strain NP-1^ T^, as well as *P. graminis* DSM11363^T^, *P. lutea* LMG21974^T^, and *P. rhizosphaerae* LMG21640^T^, failed to produce fluorescent pigments after growth for 24–48 h at 25 °C on King B medium. The tested type strains with the exception of *P. coleopterorum* LMG 28558^ T^, exhibited positive oxidase activity. Similar to *P. coleopterorum* LMG28558^T^, *P. graminis* DSM11363^T^ and *P. lutea* LMG21974^T^, the NP-1^ T^ nitrate reduction is negative. In the Biolog GN2 plates, NP-1^ T^ utilized dextrin, glycogen, L-arabinose, D-fructose, D-galactose, gentiobiose, α-D-glucose, D-mannose, D-psicose, L-rhamnose, D,L-lactic acid, quinic acid, succinic acid, bromo, succinic acid, succinamic acid, glucuronamide, L-aspartic acid, D-trehalose, formic acid, D-galacturonic acid, D-gluconic acid, D-glucuronic acid. And α-keto glutaric acid, D-saccharic acid, L-alaninamide, L-asparagine, hydroxy-L-proline, turanose, methyl pyruvate, cis-aconitic acid, D-galactonic acid lactone tests were variable. Other tests were negative in the Biolog GN2 plate. The differential phenotypic characteristics in the Biolog GN2 test are indicated in Table 3; starch hydrolysis reaction was positive in NP-1^ T^, but negative in *P. gramis* and *P. rhizosphaerae*; NP-1^ T^ can use D-sorbitol, which is different from *P. gramis*, *P. lutea* and *P. rhizosphaerae*; NP-1^ T^ could not utilize propionate, but *P. coleopterorum*, *P. gramis* and *P. rhizosphaerae* could.

**Table 3:** Phenotypic characteristics distinguishing *Pseudomonas eucalypticola* sp. nov. from phylogenetically related *Pseudomonas* type strains. 1, *Pseudomonas eucalypticola* NP-1^T^; 2, *P. coleopterorum* LMG28558^T^; 3, *P. graminis* DSM11363^T^; 4, *P. lutea* LMG21974^T^; and 5, *P. rhizosphaerae* LMG21640^T^.

Characteristics	1	2	3	4	5
Temperature ℃	4–37	4–30	4–41	10–30	10–42
Growth at 37℃	+	−	+	−	+
NaCl (%, w/v)	0–2	0–5	0–2	0–2	0–5
pH	3–7	4–10	6–8	5–7	5–9
Fluorescence	−	+	−	−	−
Oxidase	−	+	−	−	−
Nitrate reduction	−	−	−	−	+
Gelatin hydrolysis	−	−	+	+	−
Starch hydrolysis	+	+	−	+	−
Maltose	−	−	−	+	−
D-Sorbitol	−	−	+	+	+
Sucrose	−	+	−	+	−
D-Trehalose	+	−	−	+	+
Monomethyl succinate	−	w	+	−	−
Formate	+	w	+	−	−
D-Glucosaminate	+	−	+	+	−
D-Glucuronate	+	−	+	+	+
p-Hydroxy-phenylacetate	−	−	−	+	−
Propionate	−	+	+	−	+
Succinamate	+	−	+	−	−
Glycyl L-glutamate	−	+	−	−	+
D,L-Carnitine	−	+	−	−	+
Urocanate	−	−	+	+	−
Putrescine	−	−	−	+	−
2-Aminoethanol	−	−	+	−	−
D,L-a-Glycerol phosphate	−	+	w	−	w

Positive( +), negative (−), weak (w).

### Chemotaxonomic analysis

A cellular fatty acid analysis of NP-1^ T^ and various reference type strains, namely, *P*. *graminis* DSM 11363^ T^, *P*. *lutea* LMG 21974^ T^, *P. coleopterorum* LMG 28558^ T^ and *P. rhizosphaerae* LMG 21640^ T^ was performed. The results from the chemotaxonomic analyses are shown in Table 4. The major cellular fatty acids of strain NP-1^ T^ were 3-hydroxydodecanoic acid (C_12:0 3-OH_), dodecanoic acid (C_12:0_), 2-hydroxydodecanoic acid (C_12:0 2-OH_), 3-hydroxydecanoic acid (C_10:0 3-OH_), hexadecanoic acid (C_16: 0_), 17-carbon cyclopropane fatty acid (C_17:0 cyclo_), C_16:1 w6c_/C_16:1 w7c_ (Summed Feature 3) and summed Feature 8 (C_18:1 w6c_/C_16:1 w7c_). Hexadecanoic acid (C_16: 0_) was the most abundant fatty acid in all tested samples. This fatty acid profile is characteristic of strains from group I which have C_10:3 3-OH_ and C_12:0 3-OH_^19^. The cellular fatty acid profile of strain NP-1^ T^ matched that of *P. lutea.* The main difference between strain NP-1^ T^ and the reference strains is related to the presence of C_19:0 cyclo w8c_, which was was only detected in NP-1^ T^ and *P. lutea*.

**Table 4:** Cellular fatty acid composition of *Pseudomonas eucalypticola* sp. nov. and the closely related species in *Pseudomonas* genus. Species/strain: 1, *P. eucalypticola* NP-1^T^; 2, *P. coleopterorum* LMG28558^T^; 3, *P. graminis* DSM11363^T^; 4, *P. lutea* LMG21974^T^; and 5, *P. rhizosphaerae* LMG21640^T^.

	1	2	3	4	5
C_10:0 3OH_	2.95	3.04	3.68	3.45	2.91
C_12:0_	2.05	4.8	4.4	6.87	4.4
C_12:0 2OH_	4.62	2.61	2.07	3.43	2.55
C_12:0 3OH_	4.42	4.22	3.88	5.3	4.28
C_16:0_	32.25	25.77	31.67	23.22	22.64
C_17:0 cyclo_	19.01	8.55	1.75	6.39	–
C_18:0_	0.53	0.73	1.43	1.17	0.58
C_19:0 cyclo w8c_	5.9	–	–	2.18	–
Summed Feature 3	10.56	17.51	33.47	24.16	37.53
Summed Feature 8	15.44	30.95	16.29	22.19	24.01

Only results with amounts higher than 0.5% for at least one strain are presented.

Summed Feature 3 contained C16:1 w6c/C16:1 w7c; summed Feature 8 contained C18:1 w6c/C16:1 w7c.

### Antifungal activity

**Figure 5:**
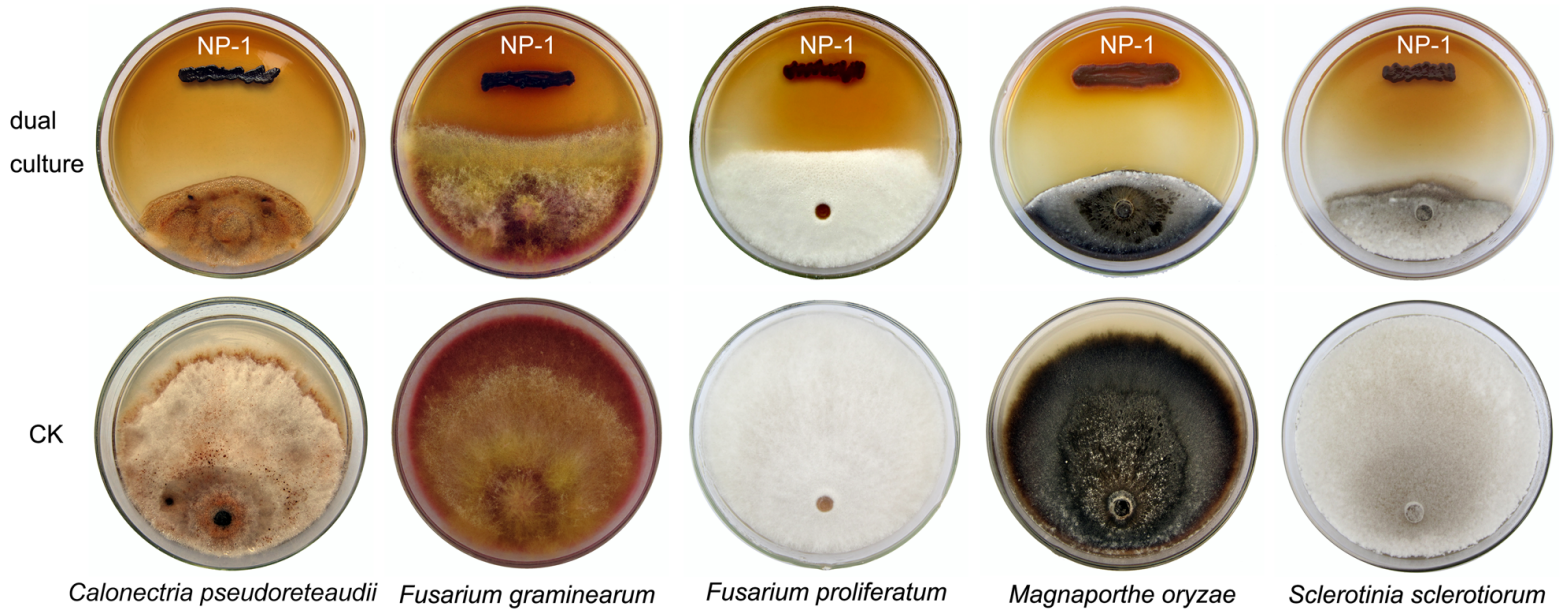
Antagonistic interaction between *Pseudomonas eucalypticola* NP-1^T^ and selected phytopathogenic fungi.

*P. eucalypticola* NP-1^ T^ exhibited antifungal activity against five tested fungal species (Fig. 5). Specifically, NP-1^ T^ exhibited strong antifungal activity against *C. pseudoreteaudii*, *M. oryzae*, and *S. sclerotiorum*, as demonstrated by the formation of an inhibition zone with a width greater than 30 mm. The contrast, the inhibition zones between NP-1^ T^ and the two *Fusarium* species had a width less than 30 mm.

## Conclusion

According to the 16S rRNA similarity and morphological results, strain NP-1^ T^ isolated from *Eucalyptus dunnii* leaves clearly belongs to the *Pseudomonas* genus. NP-1 was distinguished from closely related *Pseudomonas* species base on the genotypically and phenotypically analysis. The MLSA and GBDP results indicate that NP-1^ T^ is representative of a new species, and the ANI, GGDC, phenotypic characterization and chemotaxonomic analysis confirm this presumption. Therefore, strain NP-1^ T^ should be assigned to a novel species with the name *Pseudomonas eucalypticola* sp. nov.. In addition, strain NP-1^ T^ can inhibit 5 species phytopathogenic fungi, belonging to 4 genera, and has thus potential for plant disease biocontrol.

### Description of P. eucalypticola sp. nov.

*Pseudomonas eucalypticola,* (eu.ca.lyp.ti'cola, N.L. fem. n. *eucalypticola*), Eucalyptus, a botanical generic name; L. suff. -cola (from L. n. incola), inhabitant. Eucalyptus-dwellar, refers to Eucalyptus, the host plant from which this bacterium was isolated.

Colonies are beige in colour, round with smooth surfaces and edges on LA medium. Cells are Gram-negative, approximately 1.0 × 2.0 μm in size, and motile due to the presence of a single polar flagellum. NP-1^ T^ can grow at temperatures ranging from 4 °C to 37 °C, in the presence of 0–2% (w/v) NaCl and at pH values of 3–7. The optimal temperature, pH and salinity for NP-1^ T^ growth are 25 °C, 6.0 and 0.5% NaCl, respectively. Oxidase, nitrate reduction and gelatin hydrolysis were negative. Strain NP-1^ T^ can oxidase a variety of carbon sources: dextrin, glycogen, L-arabinose, D-fructose, D-galactose, gentiobiose, α-D-glucose, D-mannose, D-psicose, L-rhamnose, D,L-lactic acid, quinic acid, succinic acid, bromo, succinic acid, succinamic acid, glucuronamide, L-aspartic acid, D-trehalose, formic acid, D-galacturonic acid, D-gluconic acid, and D-glucuronic acid. Weak positive reactions were obtained for α-keto glutaric acid, D-saccharic acid, L-alaninamide, L-asparagine, hydroxy-L-proline, turanose, methyl pyruvate, cis-aconitic acid, D-galactonic acid lactone test. NP-1^ T^ does not oxidize the other organic substrates.

The most abundant FAMEs are C_16:0_, C_17:0 cyclo_, Summed Feature 8, and Summed Feature 3. The G + C mol % base composition is 63.96, and the type strain is NP-1^ T^ (= CCTCC M2018494^T^ = JCM 33572^ T^).

## Methods

### Bacterial and fungal strains and growth conditions

Strain NP-1^ T^ has been isolated from healthy *Eucalyptus dunnii* leaves which were collected at Xiayang Town, Nanping, Fujian Province (26°46′14.9"N 118°00′21.1"E). As reference strains, the type strains of *P*. *coleopterorum* LMG28558^T^^20^, *P*. *lutea* LMG21974^T^^21^ and *P. rhizosphaerae* LMG21640^T^(= DSM 16299^T^)^22^ were purchased from BCCM/LMG Bacteria Collection, Belgium, and *P*. *graminis* DSM11363^T^^23^ were from Leibniz-Institut DSMZ, Germany. All bacteria were cultured at 28℃ on LA medium for 24–48 h. Five phytopathogenic fungal species, *C. pseudoreteaudii*, *Fusarium graminearum*, *Fusarium proliferatum*, *Magnaporthe oryzae*, *Sclerotinia sclerotiorum* were cultured on PDA plates at 25℃.

Lysogeny broth (LB) medium was prepared with 10 g peptone, 5 g yeast extract and 5 g NaCl in each 1000 mL deionized water, the pH was adjusted to 7.0 with l M NaOH solution; LA is LB with 15 g ager powder added. Potato Dextrose Agar (PDA) was made using the following procedure: boil 200 g of sliced unpeeled potatoes in 1 L of water for 30 min, then filter through cheesecloth, saving effluent, which is potato infusion, and add dextrose, agar, and water to effluent, Boil to dissolve completely. All the media were sterilize media by autoclaving at 121ºC for 15 min.

### PCR amplification and DNA sequencing

For DNA extraction, one bacterial colony was inoculated with 5 ml of LB media and cultured for 24 h at 28℃ in a shaker (200 rpm). Bacterial cells were harvested by centrifugation for 1 min at 10 000 × *g*. The bacterial DNA was isolated using Omega D3350-00 Bacterial DNA Kit (OMEGA Bio-tec, USA). In order to check the taxonomic placement of the NP-1^T^, near-complete 16S rRNA was amplified with primers 27F and 1492R^24^. Partial *gyrB*, *rpoB* and *rpoD* gene were amplified using primers gBMM1F/ gBMM725R for *gyrB* fragment^25^, LAPS/ LAPS27 for *rpoB* fragment^26^ and PsEG30F/PsEG790R for *rpoD* fragment^27^. All PCR production were sequenced and deposited in GenBank. PCR amplification was performed with a DNA thermocycler (Bio-Rad, T1000). Each reaction mixture contained 25 μl 2 × EasyTaq PCR superMix (TSE030, Tsingke Biotech), respective 1 μl of each primer (10 μM) and 1 μl DNA template in a total volume of 50 μl. The amplified products were purified with EasyPure Quick Gel Extraction Kit (CE101-01, TransGen Biotech, China), cloned and sequenced using the procedures described by Zhang et al.^28^.

### Phylogenetic analysis

To ascertain the taxonomic position of NP-1, the 16S rRNA gene was compared against related available 16S rRNA gene sequences in the EzBioCloud database (http://www.ezbiocloud.net)^29^. The phylogenetic analysis was performed using MEGA 7 software, based on the neighbor-joining method with 1000 bootstrap replicates under the maximum composite Likelihood model^30^.

For further determination of the phylogenetic position of NP-1^T^, a multilocus sequencing analysis (MLSA) with the concatenated four genes, namely, 16S rRNA, *gyrB*, *rpoB* and *rpoD*, was also performed. The 16S rRNA (1444 bp), *gyrB* (743 bp), *rpoB* (915 bp) and *rpoD* (711 bp) gene sequences were concatenated in the following order: 16S rRNA-*gyrB*-*rpoB*-*rpoD*. This realignment resulted in a 3813-bp-long sequence. The remaining sequences included in this manuscript were obtained from public databases, and their accession numbers are listed in Supplementary table S2. The phylogenetic tree was constructed based on the concatenated sequences obtained using the above-mentioned method.

### Genome sequencing and analysis

The genome of *P. eucalypticola* NP-1^T^ was sequenced using single molecule, real-time (SMRT) technology at Beijing Novogene Bioinformatics Technology Co., Ltd. The low quality reads were filtered with the SMRT 2.3.0, and the filtered reads were assembled to generate one contig without gaps^31,32^. The DNA G + C mol % was obtained from the genomic sequences, and the G + C mol % differences between NP-1^T^ and its closely related species were calculated and cannot be larger than 1 within the same species^33^. The similarity of the sequenced genome of *P. eucalypticola* NP-1^T^ to public genomes of closely related *Pseudomonas* species was determined based on the average nucleotide identity (ANI) and tetranucleotide signatures (TETRA). The TETRA, BLASTn (ANIb) and the MUMMER ultrarapid aligning (ANIm) results were calculated using the JSpecies software tool available at http://jspecies.ribohost.com/jspeciesws with the recommended species cut-off of 95–96% for ANI and a value higher than 0.99 for the TETRA signature^34,35^. The DNA-DNA hybridization (DDH) was calculated in silico using the GGDC. Calculation of GGDC was performed at http://ggdc.dsmz.de/ webpage using the GGDC 2.1 service with the BLAST + method. GGDC results were based on recommended formula 2 (identities / HSP length), which is independent of the genome length and is thus robust against the use of incomplete draft genomes^36^. The phylogenomic tree inferred with FastME 2.1.4 from distances calculated Genome BLAST Distance Phylogeny (GBDP) was constructed using Type (Strain) Genome Server (TYGS) web servers: https://tygs.dsmz.de/18.

### Morphology, physiological and biochemical tests

Gram staining performed out according to standard methods. The cell morphology was examined by scanning electron microscopy (EVO 10, Zeiss), and flagella arrangements were determined using transmission electron microscopy (TEM) after overnight incubation in LB medium at 25 °C. A Hitachi HT7800 model TEM was used at 80 kV. The samples were negatively stained with phospho-tungstic acid (1%, pH 7.0) as previously described^37^. The fluorescent pigment was observed on King medium B^16^. The growth at various temperatures (4, 10, 25, 28, 30, 37, 38, 39, 40, 41 and 42 °C) was investigated over 5 days, and growth was assessed based on the occurrence of visible colonies on LA plates. The growth in the presence of salt (NaCl, 0, 0.5 and 1–10% w/v) and at pH values range (1–14) was tested using LB media by monitoring the OD_600_ changes after 2 days. The pH was adjusted using adding sterilized 1 M NaOH and HCl solution. Hydrolysis of gelatin was investigated using microbiochemical bacterial identification tubes (GB191, Hopebio). The experiments above were repeated twice. Additional physiological and biochemical characteristics were determined using the GN2 Biolog microplate and API 20NE system according to the manufacturer’s instructions.

### Chemotaxonomic analysis

Whole cell fatty acid methyl esters (FAME) of *P. eucalypticola* NP-1^T^ and all the reference type strains were studied at the Guangdong Culture Collection, Guangzhou, China under standardized conditions. The methods used for the harvesting, saponification, methylation and extraction of cellular fatty acids followed the protocols detailed by the Sherlock Microbial Identification System (MIDI). The cellular fatty acid peaks, names and percentages were analyzed using an Agilent 6890 N gas chromatograph, with the MIDI Microbial Identification System using the TSBA6 method and the Sherlock Microbial Identification software package version 6.1^38^.

### Antifungal test

Dual cultures in petri dishes were used to detect the antifungal activity of *P. eucalypticola* NP-1^T^. Five phytopathogenic fungal species, namely, *C*. *pseudoreteaudii*, *F*. *graminearum*, *F*. *proliferatum*, *M*. *oryzae*, and *S*. *sclerotiorum* were selected for testing. These five plant pathogen fungi are distributed all over the world, causing a large number of deaths of eucalyptus trees, wheat, oilseed rape and rice each year, threatening the development of agroforestry. A loop of NP-1^T^ cells was streaked on a PDA dish 1 cm from the edge. A mycelial agar plug (6 mm in diameter) of each fungus obtained from the margin of the colony was placed 5 cm from the NP-1^T^ inoculant to establish a dual culture. Five dishes (replicates) of each fungus were used, and the cultures were incubated at 25 °C. The clear zone that formed between NP-1^T^ and the fungal plug in each dish was considered an indicator of the antifungal capability of NP-1^T^. The inhibition zone width was measured after 72 h of incubation.

## Supplementary Information

Supplementary Files
