# Supplementary material for: Pseudomonas eucalypticola sp. nov., a producer of antifungal agents isolated from Eucalyptus dunnii leaves
Source: Sci Rep. 2021 Feb 4;11:3006. doi: 10.1038/s41598-021-82682-7 (PMC7862484; doi:10.1038/s41598-021-82682-7)
Supplement: Supplementary file 1 — Supplementary Files [file 41598_2021_82682_MOESM1_ESM.docx]

***Pseudomonas eucalypticola* sp. nov., a producer of antifungal agent isolated from** ***Eucalyptus dunnii* leaves**

Yujing Liu, Zhang Song, Hualong Zeng, Meng Lu, Weiyao Zhu, Xiaoting Wang, Xinkun Lian, Qinghua Zhang*

Institute of Forest Protection in Forestry College of Fujian Agriculture and Forestry University, Fuzhou, 350002, China

***Corresponding author**

Qinghua Zhang

Email: [zhangqinghua@fafu.edu.cn](mailto:zhangqinghua@fafu.edu.cn)

**Supplementary materials**

**Table S1** Closest related species of Pseudomonas strain NP-1^T^ based on 16S rRNA similarity (>97%)*

| Taxon name | Strain name | Accession | Similarity |
| --- | --- | --- | --- |
| *Pseudomonas vancouverensis* | ATCC 700688^T^ | AJ011507 | 98.8 |
| *P. moorei* | RW10^T^ | AM293566 | 98.8 |
| *P. koreensis* | Ps 9-14^T^ | AF468452 | 98.8 |
| *P. parafulva* | NBRC 16636^T^ | BBIU01000051 | 98.5 |
| *P. reinekei* | Mt-1^T^ | AM293565 | 98.5 |
| *P. moraviensis* | CCM 7280^T^ | AY970952 | 98.4 |
| *P. jessenii* | DSM 17150^T^ | NIWT01000013 | 98.4 |
| *P. mohnii* | DSM 18327^T^ | FNRV01000001 | 98.3 |
| *P. umsongensis* | DSM 16611^T^ | NIWU01000003 | 98.3 |
| *P. fulva* | NBRC 16637^T^ | BBIQ01000036 | 98.3 |
| *P. baetica* | a390^T^ | FM201274 | 98.1 |
| *P. granadensis* | LMG 27940^T^ | LT629778 | 98.2 |
| *P. soli* | F-279,208^T^ | HF930598 | 97.8 |
| *P. graminis* | DSM 11363^T^ | Y11150 | 97.8 |
| *P. lutea* | LMG 21974^T^ | AY364537 | 97.8 |
| *P. plecoglossicida* | NBRC 103162^T^ | BBIV01000080 | 97.7 |
| *P. kuykendallii* | NRRL B-59562^T^ | JF749828 | 97.7 |
| *P. monteilii* | NBRC 103158^T^ | BBIS01000088 | 97.7 |
| *P. rhizosphaerae* | DSM 16299^T^ | CP009533 | 97.7 |
| *P. putida* | NBRC 14164^T^ | AP013070 | 97.7 |
| *P. entomophila* | L48^T^ | CT573326 | 97.7 |
| *P. turukhanskensis* | IB1.1^T^ | KP306892 | 97.4 |
| *P. helmanticensis* | OHA11^T^ | HG940537 | 97.6 |
| *P. mosselii* | CIP 105259^T^ | AF072688 | 97.7 |
| *P. punonensis* | CECT 8089^T^ | FRBQ01000018 | 97.6 |
| *P. reidholzensis* | CCOS 865^T^ | LT009707 | 97.6 |
| *P. taiwanensis* | BCRC 17751^T^ | EU103629 | 97.9 |
| *P. coleopterorum* | Esc2Am^T^ | KM888184 | 97.7 |
| *P. donghuensis* | HYS^T^ | AJJP01000212 | 97.4 |
| *P. alkylphenolica* | KL28^T^ | CP009048 | 97.4 |

*the data from EzBioCloud 16S database with valid names only.

**Table S2** Accession numbers of the sequences of the type strains of different *Pseudomonas* species used in the MLSA phylogenetic analysis.

| Species | Type strain | Gene and Accession number | | | |
| --- | --- | --- | --- | --- | --- |
|  |  | 16S | *gyrB* | *rpoB* | *rpoD* |
| *P. eucalypticola* | NP-1^T^ | MN238862 | MN 233594 | MN 233595 | MN 233596 |
| *P. coleopterorum* | Esc2Am ^T^ | KM888184 | FNTZ010000011:4674081-4676604 | KM888186 | KM888187 |
| *P. graminis* | DSM 11363 ^T^ | Y11150 | FN554187 | AJ717429 | ,FN554469 |
| *P. kuykendallii* | NRRL B-59562 ^T^ | JF749828 | LT615253 | FNNU01000011:5421-9555 | LMG 26364 |
| *P.* *lutea* | LMG 21974 ^T^ | AY364537 | FN554198 | FN554738 | FN554480 |
| *P. mohnii* | DSM 18327 ^T^ | AM293567 | AM293561 | FN554741, | FN554487 |
| *P. moorei* | DSM 12647 ^T^ | AM293566 | TAM293560 | FN554742 | FN554489 |
| *P.* *rhizosphaerae* | DSM 16299 ^T^ | CP009533 | FN554224 | FN554755 | FN554510 |
| *P. umsongensis* | LMG 21317 ^T^ | NR025227 | FN554231 | FN554763 | FN554516 |
| *P. vancouverensis* | ATCC 00688^T^ | AJ011507 | FN554232 | NZ_LT629803:6114764-6115679 | FN554517 |
| *Cellvibrio japonicus* | Ueda107 ^T^ | NC010995:807088-808555 | NC010995:3980-6400 | NC010995:821813-825898 | NC010995:895987-896726 |
| *P. fluorescens* | ATCC 13525 ^T^ | D84013 | D86016 | AJ717451 | AB039545 |
| *P. gessardii* | LMG 21604 ^T^ | AF074384 | FN554186 | AJ717438 | FN554468 |
| *P. meridiana* | CIP 108465 ^T^ | AJ537602 | FN554203 | FN554740 | FN554485 |
| *P. fragi* | CECT 446 ^T^ | AF094733 | FN554184 | AJ717444 | FN554466 |
| *P. mandelii* | LMG 21607 ^T^ | AF058286 | FN554200 | AJ717435 | FN554482 |
| *P. deceptionensis* | CECT 7677 ^T^ | GU936597 | HE800476 | HE800510 | GU936596 |
| *P. arsenicoxydans* | CECT 7543 ^T^ | FN645213 | FN645139 | HE800503 | FN645160/HE800488 |
| *P. koreensis* | LMG 21318 ^T^ | AF468452 | FN554194 | FN554737 | FN554476 |
| *P. corrugata* | ATCC 29736 ^T^ | D84012 | AB039460 | AJ717487 | AB039566 |
| *P. mediterranea* | CFBP 5447 ^T^ | AF386080 | AM084678 | AJ717449 | AM084337 |
| *P. chlororaphis* subsp. *aureofaciens* | DSM 6698 ^T^ | AY509898 | FN554172 | AJ717426 | FN554453 |
| *P. asplenii* | LMG 2137 ^T^ | AB021397 | AB039455 | AJ717432 | AB039593 |
| *P. fuscovaginae* | LMG 2158 ^T^ | FJ483519 | FN554185 | AJ717433 | FN554467 |
| *P. syringae* | NCPPB 281 ^T^ | DQ318866 | AB039428 | FN554759 | AB039516 |
| *P. abietaniphila* | ATCC 700689 ^T^ | AJ011504 | FN554166 | AJ717416 | FN554447 |
| *P. monteilii* | ATCC 700476 ^T^ | AF064458 | FN554205 | AJ717455 | FN554488 |
| *P. putida* | ATCC 12633 ^T^ | D84020 | AB039451 | AJ717474 | AB039581 |
| *P. soli* | LMG 27941 ^T^ | HF930598 | LN851840 | HF930596 | HF930597 |
| *P. taiwanensis* | DSM 21245 ^T^ | EU103629 | FJ418634/HE800487 | HE577797 | HE577796 |
| *P. straminea* | LMG 21615 ^T^ | D84023 | AB039410 | FN554758 | AB039600 |
| *P. flavescens* | LMG 18387 ^T^ | U01916 | FN554183 | AJ717468 | FN554465 |
| *P. benzenivorans* | DSM 8628 ^T^ | FM208263 | HE800472 | HE800506 | HE800490 |
| *P anguilliseptica* | LMG 21629 ^T^ | X99540 | FN554168 | AJ717417 | FN554449 |
| *P peli* | LMG 23201 ^T^ | AM114534 | FN554217 | FN554750 | FN554501 |
| *P guineae* | M8 ^T^ | AM491810 | FN554189 | FN554734 | FN554471 |

**Table S3** Type strains and their genome sources in the phylogenomic tree

| Species | Type strain | Genome source |
| --- | --- | --- |
| *Pseudomonas eucalypticola* | NP-1^T^ | CP056030 |
| *Pseudomonas* *aeruginosa* | DSM 50071^T^ | GCA_900167195 |
| *Pseudomonas* *anguilliseptica* | DSM 12111^T^ | GCA_900105355 |
| *Pseudomonas* *coleopterorum* | LMG 28558^T^ | GCA_900105555 |
| *Pseudomonas* *fluorescens* | DSM 50090^T^ | GCA_001269845 |
| *Pseudomonas fulva* | DSM 17717 | GCA_000621265 |
| *Pseudomonas* *graminis* | DSM 11363^T^ | GCA_900111735 |
| *Pseudomonas* *jessenii* | DSM 17150^T^ | GCA_002236115 |
| *Pseudomonas koreensis* | LMG 21318^T^ | GCF_900101415 |
| *Pseudomonas* *kuykendallii* | NRRL B-59562^T^ | GCA_900106975 |
| *Pseudomonas* *lutea* | DSM 17257^T^ | GCA_000759445 |
| *Pseudomonas mohnii* | DSM 18327^T^ | GCA_900105115 |
| *Pseudomonas moorei* | DSM12647^T^ | GCF_900102045 |
| *Pseudomonas moraviensis* | LMG 24280^T^ | GCF_900105805 |
| *Pseudomonas parafulva* | DSM 17004^T^ | GCA_000425765 |
| *Pseudomonas* *putida* | NBRC 14164^T^ | GCA_000412675 |
| *Pseudomonas reinekei* | MT1^T^ | GCA_001945365 |
| *Pseudomonas* *rhizosphaerae* | DSM 16299^T^ | GCA_000761155 |
| *Pseudomonas umsongensis* | DSM 16611^T^ | GCA_002236105 |
| *Pseudomonas vancouverensis* | LMG 20222^T^ | GCF_900105825 |


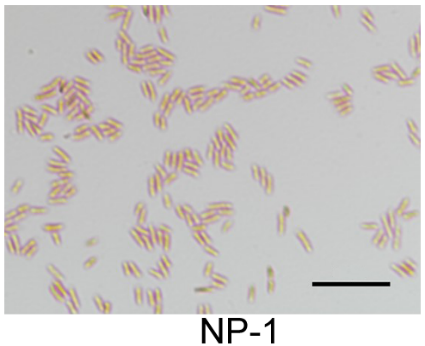


Fig. S1 Gram staining of Pseudomonas *eucalypticola* NP-1^T^
